# Comparing emergency department versus high school-based recruitment for a hypertension research study with adult–youth dyads

**DOI:** 10.1017/cts.2024.586

**Published:** 2024-09-16

**Authors:** Sara W. Heinert, Ryan Salvatore, Kelsey M. Thompson, Divya Krishna, Kayla Pena, Pamela Ohman-Strickland, Kathryn Greene, Carolyn J. Heckman, Benjamin F. Crabtree, Phillip Levy, Shawna V. Hudson

**Affiliations:** 1 Department of Emergency Medicine, Rutgers Robert Wood Johnson Medical School, Rutgers University, New Brunswick, NJ, USA; 2 Rutgers Robert Wood Johnson Medical School, New Brunswick, NJ, USA; 3 Department of Emergency Medicine, Harbor-UCLA Medical Center, Torrance, CA, USA; 4 Department of Emergency Medicine, Penn Medicine, Philadelphia, PA, USA; 5 Department of Biostatistics and Epidemiology, Rutgers School of Public Health, Piscataway, NJ, USA; 6 Department of Communication, Rutgers University, New Brunswick, NJ, USA; 7 Rutgers Cancer Institute, Rutgers University, New Brunswick, NJ, USA; 8 Department of Family Medicine and Community Health, Rutgers Robert Wood Johnson Medical School, Rutgers University, New Brunswick, NJ, USA; 9 Department of Emergency Medicine, Wayne State University, Detroit, MI, USA

**Keywords:** Hypertension, adolescents, recruitment, emergency department, research methods, dyadic research, community engagement

## Abstract

Dyads can be challenging to recruit for research studies, but detailed reporting on strategies employed to recruit adult–adolescent dyads is rare. We describe experiences recruiting adult–youth dyads for a hypertension education intervention comparing recruitment in an emergency department (ED) setting with a school-based community setting. We found more success in recruiting dyads through a school-based model that started with adolescent youth (19 dyads in 7 weeks with < 1 hour recruitment) compared to an ED-based model that started with adults (2 dyads in 17 weeks with 350 hours of recruitment). These findings can benefit future adult–youth dyad recruitment for research studies.

## Introduction

Inclusion of a support person in behavioral interventions for chronic illness has shown to be more impactful than usual medical care or intervention involving only the patient [1,2]. However, dyads can be challenging to recruit for research studies because both people must meet eligibility criteria and be willing to participate. Behavioral intervention trials involving patients and a support person rarely report strategies employed to recruit support persons in the study [3]. Additionally, much of the literature that presents methodological reflection on the recruitment of dyads for health-related research has been composed of two adults (one patient and one support person) [4–7] or one younger child and an adult [8–11] and not adolescent-adult dyads.

Hypertension (HTN) affects a large portion of the population [12], and uncontrolled HTN is especially prevalent for underserved groups [13,14]. A youth-led HTN education intervention can warrant benefits to both hypertensive adults and youth themselves. For adults, a lack of HTN knowledge is a common barrier to HTN control, while social support is a strong facilitator [16,17]. Youth have shown increased self-confidence when given the responsibility to provide health education and care navigation to others [18,19] and can also expand their own knowledge and increase their healthful decision-making capacity [19,20]. As such, we planned a RCT of adults with HTN who present to the emergency department (ED) and youth (15–18 years) with whom they live (adult–youth dyads) to measure the effectiveness of a youth-led digital HTN education intervention to improve adult blood pressure (BP) and adult and youth HTN knowledge. We targeted adults in the ED because the underserved populations [15] seen disproportionately in the ED tend to have higher rates of uncontrolled BP [13].

Given the challenges of recruiting dyads, and especially dyads including adolescents, this manuscript describes experiences and lessons learned in recruiting adult–youth dyads for a digital HTN education intervention using recruitment in a clinical ED setting compared to a subsequent shift to recruitment in a community high school setting.

## Methods

### Intervention

The youth-led HTN education digital intervention is an electronic tool to guide youth through learning and then teaching and encouraging adults about HTN and how to achieve better HTN control. The intervention contained six weekly online modules housed in an online system (REDCap), each with a theme (e.g., healthy eating). After reviewing the educational material, both the adult and youth complete a HTN activity and submit evidence of completion for each module.

### ED recruitment (initial recruitment phase)

In August 2022, we began recruitment for a two-arm randomized controlled trial (RCT) to test the effectiveness of the intervention. A research assistant (RA) determined initial eligibility by checking the adult ED patient’s electronic health record (EHR) for two BP readings ≥ 130/80 mmHg during their ED visit. The RA then approached initially eligible patients to ask them additional eligibility questions: (1) having a history of HTN and (2) having an existing relationship (e.g., family member) with a youth (15–18-year-old who lives in the same home), and (3) speaking English or Spanish. For eligible and interested patients, the RA obtained the participant’s written consent, BP, contact information, and a brief baseline assessment. The participant received a study flier to bring home to the potential youth participant asking the youth to contact the study team if they are interested in participating. If the team did not hear from the youth after 3 days, they contacted the participant to follow up about the youth. For the dyad to participate, both adult and youth had to agree to participate and complete appropriate consent documents, including a parental permission form for minor youth. Dyads were randomized to the intervention or control arm. One week and 2 months after the 6-week intervention period, adults in both arms were to return to the hospital for follow-up BP check and to complete follow-up assessments. Youth completed follow-up assessments remotely at the same time points. Pre- and post-assessments measured BP (adults), HTN knowledge, confidence in managing high BP (youth), BP self-care activities (adults), and self-efficacy to manage HTN (adults). Youth and adult participants each received a $20 gift card for completing assessments at each time point and adults received parking or public transportation passes to return.

After several weeks of attempted study recruitment, we made modifications to address recruitment issues. First, we encountered many ED patients who could not identify youth who met the inclusion criteria of 15–18 years old and living with the adult. Thus, 2 months after recruitment began (10/25/22), we expanded youth eligibility to 14–24 years old without the need to live with the adult. Second, few youths contacted the study team after receiving the recruitment flier from the enrolled adult, so we modified the protocol 2.5 months after recruitment began (11/17/22) to allow adults (if agreeable) to share youth contact information so we could contact the youth directly. In December 2022, we revised the protocol to allow for texting of study participants in addition to phone calls and emails. All protocol changes were implemented following IRB approval.

### High school recruitment

Due to challenges recruiting youth through adults in the ED, we pivoted to a different recruitment method that began with interested youth from a local health sciences high school with which the principal investigator (PI) had an existing collaborative relationship. To recruit youth, we posted recruitment fliers at the school, and the PI shared a school-wide message during morning announcements via Google Meet. During this time, teachers could put any student questions in the chat feature so that the PI could answer them. All students at the school were asked to pair themselves with an interested adult with whom they had an existing relationship and could speak their preferred language (English or Spanish), preferably one with HTN (but not required), and with whom the student could complete an online module every week for 6 weeks. With the inclusion of adults without HTN, we expanded our focus to both controlling (adults with HTN) and preventing (adults without HTN) HTN.

Interested students contacted the study team and were asked to complete a brief REDCap form with their contact information. The study team then sent the youth a recruitment flier to share with their adult. Adults interested in participating completed a REDCap form with their contact information so that a RA could contact them to set up a time to learn about the study, ask questions, and complete consent forms. Adults could complete consents at the school, but all were completed via Zoom. Although not required to be a parent, all adults were parents/guardians of the youth, so they also completed the parental permission form. After consenting, adults completed their baseline assessments in REDCap.

After the adult was consented, the study team met with participating students at the school during their advisory/homeroom period for consenting, completion of baseline assessments, and intervention training in three rolling cohorts. Students then completed the 6-week digital educational modules with their adult at home. Each participant (adult and youth) completed post-assessments 1 week and 1 month after the end of the 6-week intervention and received a $20 gift card for each. Outcomes remained the same for this recruitment method, except we did not collect BP, since adults were not required to be diagnosed with HTN, and self-efficacy to manage HTN was only collected for adults with HTN. The study was approved by the Rutgers University Institutional Review Board.

## Results

### ED recruitment metrics

**Figure 1.**
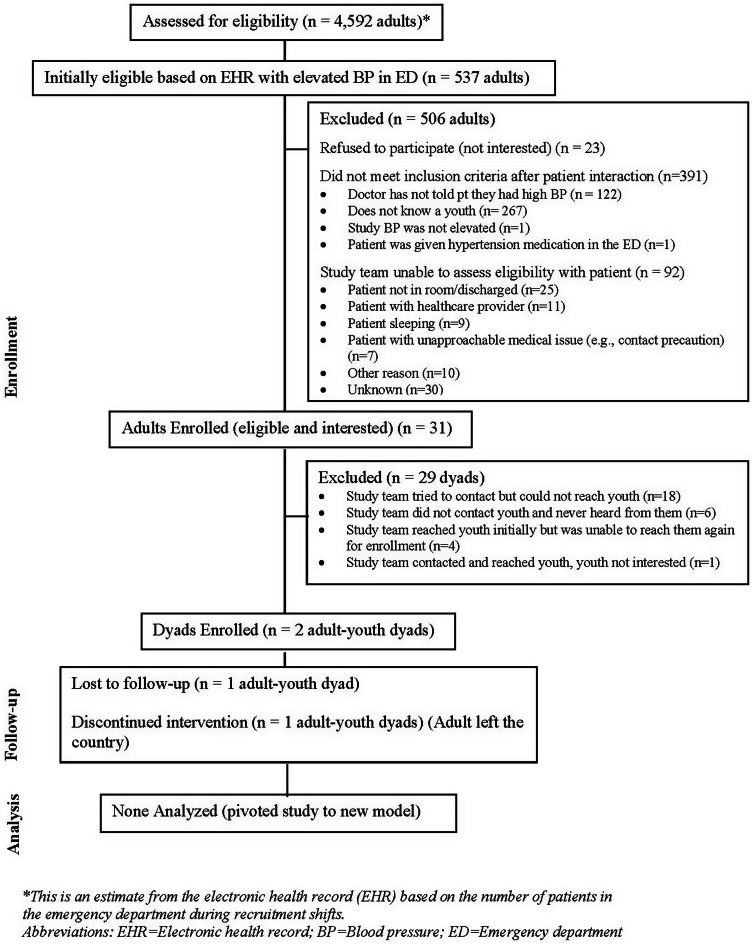
Emergency department recruitment CONSORT diagram.

We conducted active recruitment in the ED for 17 weeks from August 26, 2022, to January 10, 2023 (with a holiday break) and spent 350.5 hours total. Figure 1 shows ED recruitment metrics. There were 537 adults initially eligible based on elevated BP in the EHR out of 4,592 total adult ED patients. Five-hundred six (506) patients were subsequently excluded. Of these, 391 did not meet inclusion criteria after patient interaction, most of whom (267) did not know a youth. Of 31 patients eligible and interested in participating, only 2 dyads enrolled. For the majority (18) of the remaining dyads (29), we tried unsuccessfully to contact the youth. The dyad that did enroll in the study was randomized to the control arm, and the youth completed the first two control modules before being lost to follow-up. The adult attended the first in-person follow-up visit but not the second.

### High school recruitment metrics

**Figure 2.**
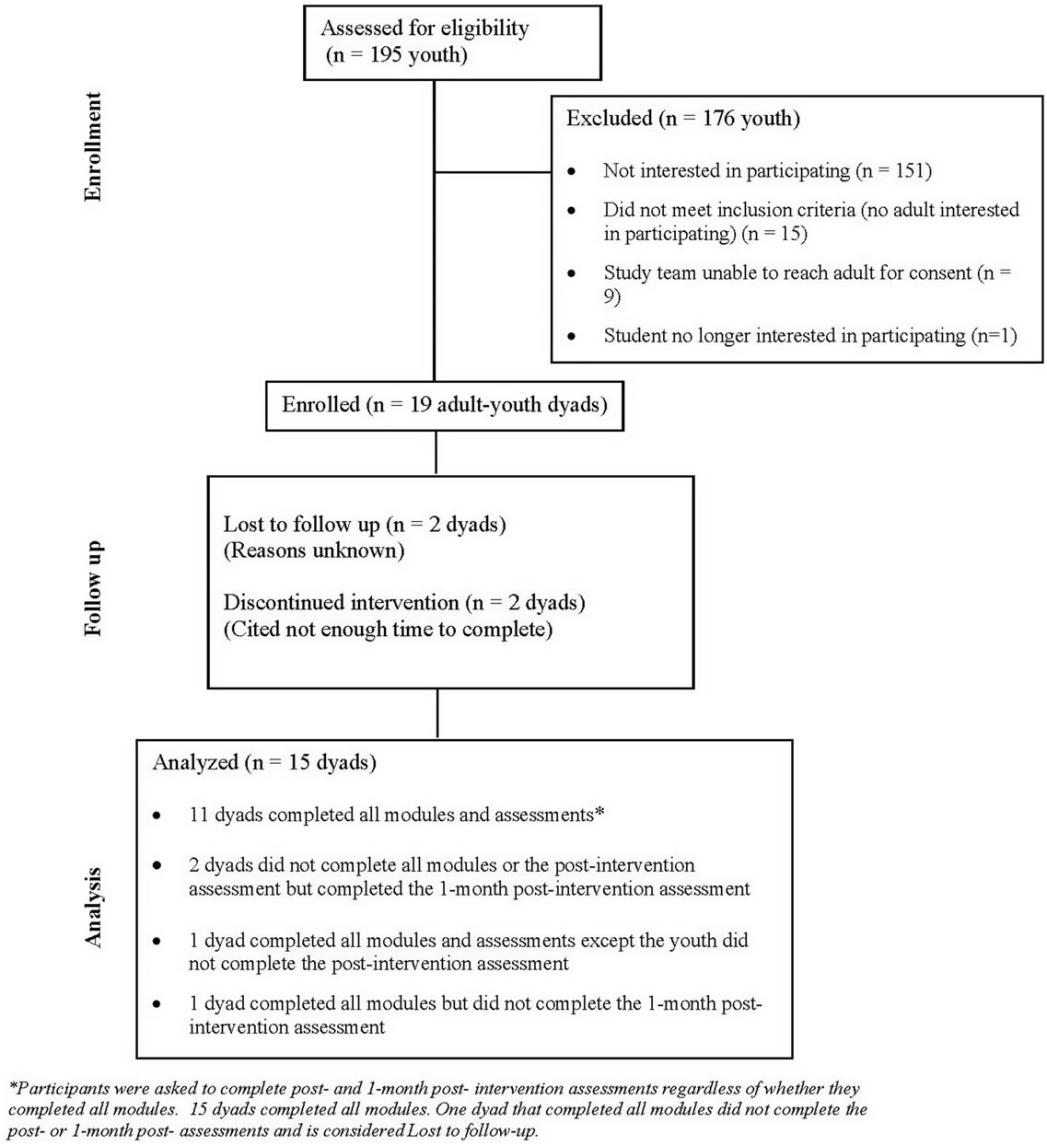
High school recruitment CONSORT diagram.

Active recruitment at the school lasted approximately 15 minutes, and the enrollment period lasted 7 weeks – February 9 to March 31, 2023. Figure 2 outlines school recruitment metrics. Of the 195 students, 44 expressed interest in participating. Of interested students who did not enroll, 15 had no interested adult, and for 9, we were unable to reach the adult for consent. Ultimately 19 dyads enrolled in the study, of which 2 discontinued the intervention due to a lack of time, and 2 were lost to follow-up for unknown reasons. Table 1 compares ED and school recruitment metrics.

**Table 1.** Emergency department and high school recruitment metrics

Recruitment location	Member of dyad approached first	Adult inclusion criteria	Youth inclusion criteria	Time spent actively recruiting for study*	Weeks spent recruiting^	Number of dyads enrolled	Number of dyads completing intervention
Emergency department	Adult	(1) Has history of hypertension (HTN) (2) Has an existing relationship (e.g., family member) with a youth (15–18-year-old) who lives in the same home^#^(3) Speaks English or Spanish	15–18-year-old who lives in the same home as adult^#^	350.5 hours	17 weeks	2 dyads	0 dyads
High school	Youth	(1) Preferably has HTN (but not required), (2) Has an existing relationship with youth (3) Speaks same language as youth (English or Spanish)	Student at the school	15 minutes	7 weeks	19 dyads	15 dyads

* This metric includes active recruitment of the first member of the dyad. We did not measure the active recruitment of the second member of the dyad or the time contacting participants about consent.

^ This metric includes active recruitment and enrollment, including obtainment of participant consent.

# Changed to: 14-24-year-old (not required to live with adult).

## Discussion

We found more efficient recruitment of adult–youth dyads through a school-based strategy that started with interested youth than through an ED-based strategy that started with interested adults. Our greatest recruitment challenges were finding adults in the ED with an existing relationship with a youth, and for those with a youth, it was difficult reaching the youth.

Several considerations likely contributed to the difference in adult–adolescent dyad recruitment success in the two models. In the ED model, we relied on the adult patient, who was already in a vulnerable position seeking healthcare in the ED, to share information with the youth, and we relied on the youth to contact us. For the school model, we collaborated with the school on recruitment methods and engaged participants in a familiar location. Also, consenting youth in-person at the school for consenting lessened their burden and may have helped with student recruitment by piquing the interest of other students who joined later during the enrollment period.

In the ED model, the adult was the first member of the dyad approached for the study and may have been more enthusiastic about participating than their youth partner. In the school model, we began with the youth, who may have been more enthusiastic to participate than their adult pair. Yet adults may have been more willing to subsequently participate if a youth was initially interested than vice versa.

Although we have compared the metrics of the two recruitment sites and methods, it is important to note limitations to this comparison. First, school recruitment included all adults – regardless of whether they had HTN, whereas we only included adults with HTN during ED recruitment. Thus, broader eligibility criteria made more people eligible for school recruitment. Additionally, this required us to modify our study outcomes for school recruitment. Because adults were not required to be diagnosed with HTN, we did not collect BP and only measured self-efficacy to manage HTN in adults with HTN. Second, the school had a health sciences focus, so may not be representative of all high schools. Third, participants recruited from the school were not randomized and all received the intervention, which may have created more interest in participating and limits our ability to measure the impact of our study’s outcomes. We also did not reach the originally targeted population of underserved adults in the ED. Lastly, unlike in the ED, adults recruited through the school were not asked for clinical data (BP) or to participate in any in-person follow-up visits, so they may have been more willing to participate.

Our findings demonstrate increased efficiency in recruiting adult–adolescent dyads for a digital HTN education intervention after shifting from an ED model to a school-based model. Researchers should consider leveraging the enthusiasm of youth through direct engagement at schools and other community settings rather than contacting them through less-engaged adults. Engaging adolescents in health promotion activities for adults can reach populations that face health disparities and be beneficial to both adults and youth participants, so they should not be overlooked due to recruitment challenges. Our findings can benefit future recruitment efforts and contribute to an existing gap in reported lessons learned when recruiting dyads of adults and adolescents for research studies.
